# High prevalence of Arginine to Glutamine Substitution at 98, 141 and 162 positions in Troponin I (*TNNI3*) associated with hypertrophic cardiomyopathy among Indians

**DOI:** 10.1186/1471-2350-13-69

**Published:** 2012-08-10

**Authors:** Deepa Selvi Rani, Pratibha Nallari, Singh Priyamvada, Calambur Narasimhan, Lalji Singh, Kumarasamy Thangaraj

**Affiliations:** 1 Centre for Cellular and Molecular Biology, CSIR, Uppal Road, Hyderabad, 500 007, India; 2 Department of Genetics, Osmania University, Hyderabad, India; 3 Department of Cardiology, CARE Hospitals, Hyderabad, India; 4 Genome Foundation, Hyderabad, India; 5 Banaras Hindu University, Varanasi, India

**Keywords:** *TNNI3-*Troponin I, Cardiomyopathy, SNPs, HCM, Indians, Mutations

## Abstract

**Background:**

Troponin I (*TNNI3*) is the inhibitory subunit of the thin filament regulatory complex Troponin, which confers calcium-sensitivity to striated muscle actomyosin ATPase activity. Mutations (2-7%) in this gene had been reported in hypertrophic cardiomyopathy patients (HCM). However, the frequencies of mutations and associated clinical presentation have not been established in cardiomyopathy patients of Indian origin, hence we have undertaken this study.

**Methods:**

We have sequenced all the exons, including the exon-intron boundaries of *TNNI3* gene in 101 hypertrophic cardiomyopathy patients (HCM), along with 160 healthy controls, inhabited in the same geographical region of southern India.

**Results:**

Our study revealed a total of 16 mutations. Interestingly, we have observed Arginine to Glutamine (R to Q) mutation at 3 positions 98, 141 and 162, exclusively in HCM patients with family history of sudden cardiac death. The novel R98Q was observed in a severe hypertrophic obstructive cardiomyopathy patient (HOCM). The R141Q mutation was observed in two familial cases of severe asymmetric septal hypertrophy (ASH++). The R162Q mutation was observed in a ASH++ patient with mean septal thickness of 29 mm, and have also consists of allelic heterogeneity by means of having one more synonymous (E179E) mutation at g.4797: G → A: in the same exon 7, which replaces a very frequent codon (GAG: 85%) with a rare codon (GAA: 14%). Screening for R162Q mutation in all the available family members revealed its presence in 9 individuals, including 7 with allelic heterogeneity (R162Q and E179E) of which 4 were severely affected. We also found 2 novel SNPs, (g.2653; G → A and g.4003 C → T) exclusively in HCM, and *in silico* analysis of these SNPs have predicted to cause defect in recognition/binding sites for proteins responsible for proper splicing.

**Conclusion:**

Our study has provided valuable information regarding the prevalence of *TNNI3* mutations in Indian HCM patients and its risk assessment, these will help in genetic counseling and to adopt appropriate treatment strategies.

## Background

Inherited cardiomyopathy is a disorder of ‘cardiac muscle’ associated with abnormalities of cardiac wall thickness, chamber size, contraction, relaxation, conduction and rhythm, were found to be the major cause of heart failure. It is also different from other heart disease, as it frequently affects all the age groups, including young children, adults and competitive athletes [1]. Over the last two decades, a large number of mutations have been identified in sarcomeric genes as a cause of hypertrophic and dilated cardiomyopathy. The sarcomere composed of thick and thin filaments, the thick filament is constituted mainly of myosin, and the thin filament is composed of actin, tropomyosin and troponin complex. Cardiac troponin I (*TNNI3),* the inhibitory sub-unit of troponin complex, is 6.2 kb in size and comprised of 8 exons that encode for 210 amino acids, prevents the contraction of muscle in the absence of calcium and troponin C, and expressed exclusively in cardiac tissue [2]. Troponin I has three binding sites, one for troponin T (residue 61-112), another for troponin C (residue 113-164) and the third one for actin-tropomyosin (residue 130-148; 173-181) of the thin filaments [3]. During the functioning of the contractile apparatus depolarization of muscle leads to intracellular release of calcium, which binds with troponin C. A conformational change occurs in troponin-tropomyosin complex in such a way that actin molecules can interact with myosin, resulting in muscle contraction. Several mutations leading to familial hypertrophic cardiomyopathy (*FHC)* have been identified in this gene [4-6]. Analysis of cardiac beta myosin heavy chain (*MYH7)* and myosin binding protein C (*MyBPC3)* in Indian HCM and DCM patients revealed few genetic variants associated with the disease [7,8]. Since the Indian populations are culturally and geographically highly heterogeneous; genetically we expected that they might exhibit unique set of mutations. As there is no comprehensive study on Indian population, we have analysed all the exons and the exon-intron boundries of *TNNI3* gene of Indian cardiomyopathy patients to assign its role in the etiology of cardiomyopathy among Indian populations.

## Results

**Figure 1:**
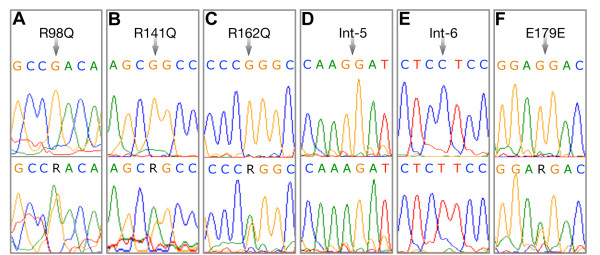
**(A - F). Sequence electropherograms of TNNI3 gene.** Upper panel representing the control sequences, whereas the lower panel showing the mutations observed in the HCM patients. The mutations sites are shown with arrows. **A**. Novel missense heterozygous mutation at the nucleotide position g.4019 (G > A) that changes the amino acid Arginine (CGA) to Glutamine (CAA) at the residue 98. **B**. A heterozygous mutation at the nucleotide position g.4682 (G > A) that changes the amino acid Arginine (CGG) to Glutamine (CAG) at residue 141. **C**. A heterozygous mutation at the nucleotide position g.4745 (G > A) that changes the amino acid Arginine (CGG) to Glutamine (CAG) at residue 162. **D**. A novel homozygous splice acceptor site SNP at the nucleotide position g.2653 (AA) in intron 5. **E**. A novel homozygous SNP at the nucleotide position g.4003 (TT) in Intron 6. **F**. A heterozygous silent mutation (E179E), at the nucleotide position g.4797 (G > A) in exon 7.

**Figure 2:**
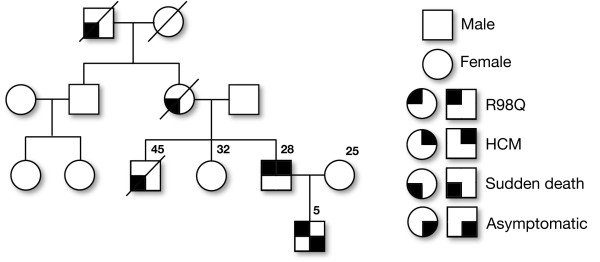
**A pedigree of a hypertrophic cardiomyopathy (HCM) family with R98Q mutation in the exon 6 of cardiac troponin I*TNNI3*gene is depicted.**

**Figure 3:**
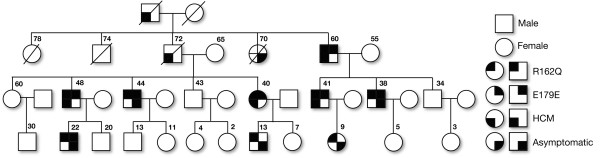
**A pedigree of a four-generation hypertrophic cardiomyopathy (HCM) family with R162Q and E179E mutations in the exon 7 of cardiac (*TNNI3)*Troponin I gene.**

Screening of all the exons including the exon-intron boundaries of the *TNNI3* gene in 101 individuals with HCM (Table1) along with 160 healthy controls from India revealed a total of 16 mutations, including 15 SNPs, and a 4 bp deletion/insertion polymorphism (Table2). Of the 15 SNPs, 7 were exonic (one novel, 3 reported non-synonymous and 3 synonymous mutations), and 9 were intronic mutations (Table2). Interestingly, we found three heterozygous arginine to glutamine (R to Q) substitution at 3 positions 98, 141 and 162 in *TNNI*3 Figure1A, 1B, 1C, of these R98Q in exon 6 of *TNNI3* gene is novel (Figure1A) observed in a 28 years old severe hypertrophic obstructive cardiomyopathy patient (HOCM) with interventricular septum (IVS) thickness of 25 mm and his 5 years old asymptomatic son, with the family history of sudden cardiac deaths (Figure2). The dominant R141Q mutation in exon 7 of *TNNI3* gene (Figure1B), lies within the “minimum inhibitory sequence” (residues 137 to 148) region, was observed in two individuals with severe familial asymmetric septal hypertrophy (ASH+), with interventricular septum (IVS) thickness of 25 and 28 mm, respectively. The dominant R162Q mutation (Figure1C) in exon 7 of *TNNI3* gene was observed in an individual with severe asymmetric septal hypertrophy (ASH) with mean thickness of 29 mm had abnormal echocardiogram/ECG. Screening for this mutation (R162Q) in all the available family members (Figure3) revealed its presence in 9 individuals (Figure1C). Seven out of 9 individuals with R162Q mutation showed allelic heterogeneity with having a synonymous mutation at g.4797: G → A: E179E (Figure1F) in exon 7, which replaces a very frequent codon (GAG: 85%) with rare codon (GAA: 14%) (Table3). Four out of 7 Individuals with allelic heterogeneity (R162Q and E179E) (Figure1C, 1F) had presented with severe septal hypertrophy (ASH++) with the mean thickness of 27, 28, 29, 32 mm and the ECGs were abnormal in all the four individuals. History of sudden cardiac death was also been recorded in this family (Figure3).

**Table 1:** Clinical features exhibited by hypertrophic cardiomyopathy (HCM) patients

**Baseline characteristics**	**(n = 101)**
Age, years	49 ± 10
Sex, males,%	62
NYHA class III, IV (%)	29
Dyspnea,%	65
Angina Pectoris,%	54
Syncope,%	33
LVESD, mm	20.3 ± 3.7
LVEDD, mm	36 ± 6.8
Septum, mm	21.2 ± 4.2
Abnormal ECG,%	62
Family history of HCM,%	37
Family history of SCD,%	32
Left ventricular outflow obstruction	49

NYHA - New York Heart Association; LVESD - Left ventricular end systolic dimension; LVEDD - Left ventricular end diastolic dimension; ECG - Electrocardiogram; HCM - Hypertrophic cardiomyopathy; SCD – Sudden cardiac death.

**Table 2:** **Mutations observed in troponin I (*TNNI3*) gene of the cases/controls**

**S.NO**	**Position**	**Location**	**AA Change**	**Major/Minor Allele**	**Mutations observed**		**Reported/Novel**
					**Control**	**HCM**	
1	g.1389	Intron 1	---	(T/C)	Nil	2	rs11667847
2	g.1403	Intron 1	---	(A1/G)	Nil	1	rs11671293
3	g.1215	Intron 1	C/A		Nil	1	rs3729707
4	g.1486-90	Intron 1	4b p del/In	ACAG	P	P	poly
5	g.1698	Intron 2	---	(T/C)	Nil	4	rs3729836
6	g.1810	Intron 3	---	(G/A)	Nil	3	rs3729837
7	g.1897	Intron 3	---	(G/A)	Nil	22	rs3729838
8	g.2560	Exon 5	R68R	(G/T)	Nil	4	rs3729711
9	g.2563	Exon 5	R69R	(C/A)	Nil	2	Reported
10	g.2601	Exon 5	P82R	(C/G)	1	2	Reported
11	g.2653	Intron 5	---	(G/A)	Nil	1	Novel
12	g.4003	Intron 6	---	(C/T)	Nil	2	Novel
13	g.4019	Exon 6	R98Q	G/A)	Nil	+1(S)	Novel
14	g.4682	Exon 7	R141Q	(G/A)	Nil	2	Reported to be associated with HCM
15	g.4745	Exon 7	R162Q	(G/A)	Nil	1+8 (FM)	Reported to be associated with HCM
16	g.4797	Exon 7	E179E	(G/A)	Nil	1+6 (FM)	rs3729841

AA- Amino acid, HCM-Hypertrophic cardiomyopathy, P-Polymorphic, SNP-Single nucleotide polymorphism, Nil- No mutation observed, S-Son, FM-family members.

**Table 3:** The codon usage in human cTnI (GenBank No. NM_000363) gene

**Nt. Site**	**SNP Reference**	**Loction**	**Nt. change**	**Codon Site**	**Type**	**Codons**	**aa**	**fraction**	**frequency**	**codon usage**
g.2560	rs3729711	Exon 5	G/T	68	Wild	CGG	(R)	0.24	28.436	6
					Mutant	CGT	(R)	0.04	4.739	1
g.2563	Novel	Exon 5	C/A	69	Wild	CGC	(R)	0.36	42.654	9
					Mutant	CGA	(R)	0.12	14.218	3
g.4797	rs3729841	Exon 7	G/A	179	Wild	GAG	(E)	0.857	85.308	10
					Mutant	GAA	(E)	0.143	14.218	3

C → T transition resulting in the replacement of proline with arginine (P82R) in exon 5, lies within the troponin T binding domain (61-112), was observed in 2 HCM patients and a control individual. A total of three synonymous mutations (g.2560; G > T; g.2563; C > A; g.4797; G > A) were observed exclusively in HCM patients (Tables 2, 3). The G → T mutation at g.2560 (R68R), observed in 4 HCM patients, replaces a frequent codon (CGG: 24%) with a less frequent one (CGT: 4%) (Table3). The C → A mutation at g.2563 (R69R), observed in 2 HCM patients, replaces the frequent codon (CGC: 36%) with rare codon (CGA: 12%) (Table3). The 4 bp deletion/insertion polymorphism in *TNNI3* gene was observed almost in equal frequency in both the patients and the controls, suggesting that it was not associated with HCM.

*In silico* analysis of 2 novel SNPs using Splicing Rainbow tool predicted abnormal splicing pattern (Table4) by removing or creating binding sites for hnRNPs or the SR proteins (http://www.ebi.ac.uk/asd-srv/wb.cgi?method). A novel splice acceptor site mutation at g.2653; G → A (Table4; Figure1D), was found exclusively in a HCM patient, had been predicted to abort a binding site for hnRNP.U_1_U_2_ and one more novel mutation at g.4003 C → T (Table4; Figure1E), was observed exclusively in the two severe HCM patients, revealed a drastic change in the binding sites for hnRNPs and SR proteins (2 sites in hnRNPs and 3 sites in SR proteins). The disturbed binding sites due to g.4003 C → T mutation were hnRNP-E_1_E_2_, hnRNP-I_1_I_2_, SRp20, SC35, U2AF65 (Table4; Figure1E), further emphasize its regulatory role, however it needs further investigation. In addition to the novel mutations, we have also observed SNPs reported elsewhere; rs11667847, rs3729836, rs3729837, rs11671293, rs3729838, rs3729711, rs3729841. The allele frequencies of the SNPs were comparable to the HapMap populations (http://www.HapMap.org).

**Table 4:** The hn RNPs and SR proteins binding site sequences in normal and mutant as predicted by “Splicing Rainbow” tool

**S:NO**	**SNP**	**LOCATION**	**Splicing Rainbow & the binding site sequences**		
			**Normal**		**Mutant**
**1**	**g.2653:G > A**	Intron 5	**GGATGCGAGG**	**hnRNP.U1U2**	**(Site destroyed)**
**2**	**g.4003:C > T**	Intron 6	**TCCTCCTCCA**	**hnRNP.E1E2**	**(Site destroyed)**
			**(No site)**	**hnRNP.I1 I2**	**CCACGTTCCTCTTCCAG (New site)**
			**CCTCCTCC**	**SRp20**	**CCTCTTCCA**
			**CTCCTCCA**	**SC 35**	**(Site destroyed)**
			**(No site)**	**U2AF65**	**TCTT (New site)**

Our concern is that the difference in frequencies of the alleles observed between the cases and controls are associated with disease or a difference unrelated to disease arising from underlying genetic differences between the populations from which the case and control samples were drawn: ‘population stratification’. In order to maximize our chances detecting such stratification, we genotyped cases and control samples using a panel of 50 ancestry-informative markers (AIMs) for inferring ancestry [7] and performed a principal components analysis on the data together with HapMap samples of Chinese, European, Yoruban [9], and found no significant difference in ancestry between cases and controls (Figure3). On the other hand, the Chinese, European, Yoruban, the cases/controls were making clusters among themselves, confirming that the 50 AIMs are sufficient for detecting whether or not ancestry differences along this axis are present. Thus, the population stratification along the axis was ruled out as the cause of the disease association.

## Discussion

About 2–7% mutations in *TNNI3* have been reported to be associated with hypertrophic cardiomyopathy patients from various populations [3,10]. As the data are often population specific, we investigated the prevalence of *TNNI3* mutations in 101 hypertrophic cardiomyopathy patients from southern India. We have identified R → Q mutation at 3 positions (R98Q, R141Q, R162Q) (Figure1A, 1B, 1C), of which a novel R98Q missense mutation was identified exclusively in a HOCM patient (1%) with early onset of clinical manifestation. This mutation was present at a functionally significant domain that lies within the Troponin T binding domain (61-112) (Figure1A), and was identified in a 28 year old proband and his 5 year old asymptomatic son. There were 3 premature sudden deaths (Grand father, mother and brother) in the family (Figure2), suggesting that the R98Q substitution is significantly associated with an adverse phenotype. The R141Q, R162Q mutations (Figure1B, 1C) reported to be associated with HCM in large tertiary referral center population [11] were also observed in our study. Both these mutations (R141Q, R162Q) were located in the carboxy terminal part of troponin I and the first binding site for troponin C domain and changes the net charge from +1 to 0. All the three R → Q mutations at 98, 141 and 162 were exclusively observed in the hypertrophic cardiomyopathy patients, these regions were reported to be the functionally significant domains. A dominant R141Q was observed in two HCM (2%) patients, (Figure1B). Screening of dominant R162Q and E179E mutations in exon 7 of *TNNI3* gene with the available family members revealed the presence of R162Q mutation in 9 individuals and the allelic heterogeneity (R162Q and E179E) in 7 out of 9 individuals (Figure1C, 1F). It is known that the non-random use of synonymous codons creates codon usage bias [12], and the translational speed and co-translational folding are the main factors that affect the correlation between synonymous codon usage and protein structure [13,14]. Though the significance of synonymous codon is still debatable, the 7 out of 9 individuals, who showed allelic heterogeneity (R162Q and E172E), four (out of 7) were affected more severely in the family (Figure3) therefore the role of second synonymous mutation (E172E) (Figure1F) could not be ignored.

**Figure 4:**
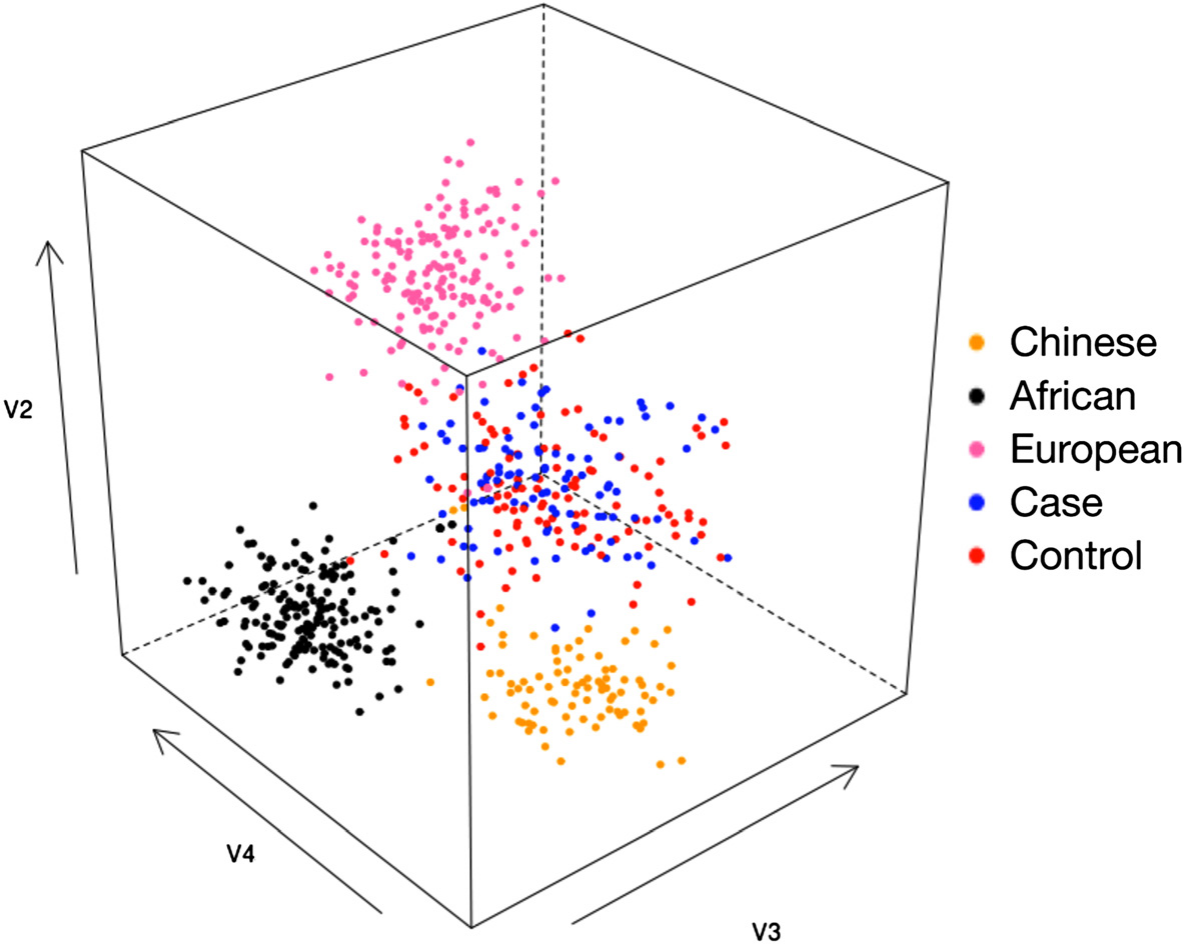
Principal components analysis of Indian case/control samples together with Chinese, African and European HapMap samples, based on a panel of 50 ancestry-informative SNP markers (AIMs).

Interestingly, two novel SNPs, one at the nt position g.2653; G → A and another one at the nt position g.4003 C → T, which were exclusively present in cases, were estimated using Splicing Rainbow tool (http://www.ebi.ac.uk/asd-srv/wb.cgi?method). In our study a splice acceptor site mutation at g.2653; G → A was found to remove a binding site for hnRNP.U_1_U_2_ and disturb the splicing (Table4; Figure1D). The g.4003 C → T mutation had revealed drastic changes at the binding sites for hnRNPs and SR proteins (hnRNP-E_1_E_2_, hnRNP-I_1_I_2_, SRp20, SC35, U2AF65), (Table3; Figure1E), thus predicted to disturb the splicing significantly. It has already been suggested that the alignment of the sequences present in the vicinity of the splice junctions has led to consensus sequences for both the splice donor and splice acceptor sites [15,16]. A panel of 50 ancestry-informative markers (AIMs) [6] for inferring ancestry was used and performed a principal components analysis on the data together with HapMap samples [9] had ruled out that these allelic differences between the cases and the controls was the cause of the disease association (Figure4). 

The frequencies of observed polymorphism in the present study were also comparable with HapMap populations. The high prevalence of R → Q mutations (4%) at three positions (R98Q, R141Q, R162Q) and the absence of remaining reported mutations observed in other populations (2-7%), is clearly elucidating the unique origin of Indian populations. These mutations (R98Q, R141Q and R162Q) can be used as markers for screening HCMs in this region. However, this study needs to be extended to know true spectrum and the prevalence of mutations present in the remaining part of the India.

## Conclusion

We have identified three R → Q mutations (R98Q, R141Q, R162Q) accounting for about 4% of HCM, of these R98Q was novel. Interestingly, two more novel SNPs, predicted to disturb the splicing significantly were also been identified in this study. Principal components analysis of the data generated by a panel of 50 ancestry-informative markers (AIMs) [6] with cases and controls for inferring ancestry together with HapMap samples had ruled out that the mutations (R98Q, R141Q, R162Q and the 2 novel SNPs (g.2653; G → A and g.4003 C → T) observed in the HCM patients was the cause of the disease association. The overall findings have provided valuable information for risk assessment, genetic counseling and adopt treatment strategies for hypertrophic cardiomyopathy among Indian patients.

## Methods

### Ethical statement

All of the DNA samples analyzed in the present study were derived from blood samples that were collected with the informed written consent of the donors. The Institutional Ethics Committee of Care Hospitals, Hyderabad and the Centre for Cellular and Molecular Biology (CSIR), Hyderabad, India, have approved the study. This study conforms to the principles outlined in the Declaration of Helsinki (WMA World Medical Association Declaration of Helsinki). The study subjects were all South Indian patients with HCM, diagnosed based on the NYHA (The Criteria Committee of the New York Heart Association, 1994), WHO (http://www.who.int/cardiovascular_diseases) guidelines for HCM; the same was applied as appropriate to rule out HCM.

### Case and control samples

Blood (about 10.0 ml) samples were collected from 101 hypertrophic cardiomyopathy patients from South India (Table1, Additional file 1: Table S1). The patients underwent physical/clinical examinations, such as; 12 lead ECGs, and trans-thoracic two-dimensional echocardiography, and Doppler studies (Table1, Additional file 1: Table S1). One hundred and sixty healthy individuals from the same ethnic background, without hypertension and cardiomyopathy, based on the electrocardiograph and echocardiograph measurements, were also recruited for the study as controls.

### DNA isolation

DNA was isolated from the blood samples using the following protocol: Erythrocytes were lysed with 15.0 ml of erythrocyte lysis buffer (10 mm Tris pH 8.0, 320 M sucrose, 5 M MgCl2, 1% Triton X-100; Sigma Chemical Company, St Louis, MO, USA) for 5 min. After complete lysis of erythrocytes, leucocytes were pelleted by centrifugation at 500 g for 5 min. The leucocyte pellet was dissolved in 8.0 ml of leucocyte lysis buffer (400 mM Tris, 60 mM EDTA, 150 M NaCl, and 1% SDS; Sigma) and mixed thoroughly. To this, 2.0 ml of 5.0 M sodium perchlorate (E. Merck, Darmstadt, Germany) was added and mixed thoroughly for 2–3 min. DNA was precipitated with absolute alcohol after extracting once with phenol:chloroform (1 : 1) and once with chloroform. DNA was washed once with 70% ethanol and dissolved in TE buffer (10 mM Tris pH 8.0, 1 mM EDTA).

### Genetic analysis

Primer sequences covering the exons, exon-intron boundaries of *TNNI3* gene, were obtained from the website (http://genepath.med.harvard.edu/~seidman/cg3/genes/TNNI3exons.html). Primers were synthesized using an ABI 392 oligo synthesizer (Perkin–Elmer, Foster City, CA, USA), and the PCRs (polymerase chain reactions) were carried out under standard conditions, containing 50 ng of genomic DNA, 5 pM of each primer, 200 mM dNTPs, 10X PCR buffer containing 1.5 mM MgCl2, and 1 unit of AmpliTaq Gold (Perkin–Elmer). Amplification was carried out in a thermal cycler (MJ Research, Waltham, MA, USA) using the following cycling conditions: 94°C for 5 min, 35 cycles at 94°C for 1 min, 55–60°C for 1 min and 72°C for 1 min, followed by a final extension at 72°C for 10 min. Amplicons were purified by treating them with ExoSAP-IT, [composed of Exonuclease 1 and Shrimp alkaline phosphatase (USB Corporation, 26, 111 Miles Road, Cleveland, Ohio 44128, USA)], according to the manufacturer’s instructions. The purified PCR products were bi-directionally sequenced using the ABI BigDye Terminator cycle sequencing kit (Perkin–Elmer, Foster City, CA, USA) and analyzed using on ABI 3730 DNA Analyzer (Applied Biosystems, Foster City, CA, USA) [17]. Sequences were edited and compared with the reference sequence (*TNNI3*) using AutoAssembler software (Applied Biosystems, Foster City, CA, USA).

### Sequenom iPLEX assay

We genotyped case/control samples for 50 Ancestry Informative Markers (AIM’s) using the Sequenom iPLEX assay where two markers failed to give results in all the individuals, therefore our final analysis was based upon 48 AIM’s. The detailed information about these markers was published elsewhere [7].

### In silico analysis

To evaluate the novel mutations observed exclusively in the hypertrophic cardiomyopathy (HCM) patients in this study was the potential cause for the defect in splicing, we have analyzed those sites with ASD Workbench wrapper (http://www.ebi.ac.uk/asd-srv/wb.cgi) tools such as PPT analysis, BP analysis. The regulatory sequence were used to identify the presence of polypyrimidine tracks, branch point sites, binding sites for splicing factors, and exonic splicing enhancers/silencers (ESE/ESS) or intronic splicing enhancers/silencers (ISE/ISS), respectively, at the mutation sites. Splicing Rainbow tool searches for the SR proteins (serine/arginine-rich) as well as hnRNP motifs were done.

## Competing interests

The authors declare that they have no competing interests.

## Authors’ contributions

DSR, PN, KT designed the study. DSR, PN carried out the blood samples collection and/or preparation of the samples. DSR performed the sequencing and statistical analysis and interpretation of the data. SP assisted with sequencing analysis. KT, PN cross-checked statistical analyses and interpretation of the data carried out by DSR. CN evaluated the patients and provided clinical information for the study. LS oversaw the data undertaken by DSR and provided the logistical support. DSR prepared the first draft of the manuscript. KT made the final interpretation of the data, and prepared the final manuscript. All authors read and approved the final manuscript.

## Pre-publication history

The pre-publication history for this paper can be accessed here:

http://www.biomedcentral.com/1471-2350/13/69/prepub

## Supplementary Material

Additional file 1 **Table S1.** Clinical phenotype of the Hypertrophic Cardiomyopathy Patients. Click here for file
