# Supplementary material for: High prevalence of Arginine to Glutamine Substitution at 98, 141 and 162 positions in Troponin I (TNNI3) associated with hypertrophic cardiomyopathy among Indians
Source: BMC Med Genet. 2012 Aug 10;13:69. doi: 10.1186/1471-2350-13-69 (PMC3495047; doi:10.1186/1471-2350-13-69)
Supplement: Additional file 1 — Table S1. Clinical phenotype of the Hypertrophic Cardiomyopathy Patients. [file 1471-2350-13-69-S1.doc]

**Table 1. Clinical features exhibited by Hypertrophic Cardiomyopathy patients (HCM)**

| **Baseline characteristics** | **(n=101)** |
| --- | --- |
| Age, Yrs | 49 ± 10 |
| Sex, Males, % | 62 |
| NYHA class III, IV (%) | 29 |
| Dyspnea, % | 65 |
| Angina Pectoris, % | 54 |
| Syncope, % | 33 |
| LVESD, mm | 20.3 ± 3.7 |
| LVEDD, mm | 36 ± 6.8 |
| Septum, mm | 21.2 ± 4.2 |
| Abnormal ECG, % | 62 |
| Family History of HCM,% | 37 |
| Family History of SCD,% | 32 |
| Left ventricular outflow obstruction | 49 |

NYHA - New York Heart Association; LVESD - Left ventricular end systolic dimension; LVEDD - Left ventricular end diastolic dimension; ECG - Electrocardiogram; HCM - Hypertrophic Cardiomyopathy; SCD – Sudden Cardiac Death.
